# Draft Genome Sequences of Two Listeria monocytogenes Strains Isolated from Invasive Snails (Arion vulgaris) in Austria in 2019

**DOI:** 10.1128/MRA.00375-21

**Published:** 2021-05-27

**Authors:** Ariane Pietzka, Andrea Murer, Anna Lennkh, Kathrin Hauser, Kornelia Vötsch, Burkhard Springer, Franz Allerberger, Werner Ruppitsch

**Affiliations:** a Austrian National Reference Laboratory for *Listeria monocytogenes*, Institute of Medical Microbiology and Hygiene, Austrian Agency for Health and Food Safety, Graz, Austria; Georgia Institute of Technology

## Abstract

We report the draft genomes of two Listeria monocytogenes strains that were isolated from the invasive alien snail species Arion vulgaris in Austria in 2019.

## ANNOUNCEMENT

Listeria monocytogenes is widespread in the environment, living as a saprophytic organism in the plant-soil compartment (1), from which it can be transferred to animals and, via contaminated food, to humans, causing the disease listeriosis. Symptoms of listeriosis can be gastroenteritis, encephalitis, meningitis, and septicemia. With a high case fatality rate of about 20% to 30%, L. monocytogenes is one of the most important foodborne human pathogens (2).

Snails have been reported recently to be important vectors for L. monocytogenes (3). To investigate the colonization of snails and to characterize environmental isolates, 45 snails of the invasive snail species Arion vulgaris were collected in diverse provinces in Austria in 2019. Detection and isolation of bacteria were performed after enrichment with Fraser bouillon, followed by plating onto selective agar according to ISO 11290-1:2017 (4). Of the 45 snails, 2 snails that had been collected in private gardens in Vienna and the province Styria were colonized with L. monocytogenes.

Genomic DNA was isolated from overnight cultures using the MagAttract high-molecular-weight (HMW) DNA kit (Qiagen, Hilden, Germany). Paired-end sequencing (2 × 300 bp) was performed with a MiSeq platform (Illumina, Inc., San Diego, CA, USA) as described (5). Library preparation was carried out using the Nextera XT kit according to the instructions of the manufacturer (Illumina, Inc.).

Default parameters were used for all software unless otherwise specified. Raw reads were quality controlled using FastQC v0.11.9. Trimmomatic v0.36 (6) was used to remove adapter sequences and to trim the last 10 bp of each sequence and sequences with a quality score of <20. Reads were assembled using SPAdes v3.11.1 (7). Contigs were filtered for a minimum coverage of 5× and a minimum length of 200 bp using SeqSphere+ v7.2.3 (Ridom GmbH, Würzburg, Germany). Assembly statistics are provided in Table 1.

**TABLE 1:** Characteristics and accession numbers of genomes of L. monocytogenes isolates from snails in Austria in 2019

Strain	Genome size (bp)	GC content (%)	No. of reads	Total no. of genes	No. of RNA genes	Avg coverage (×)	No. of contigs	Contig *N*_50_ (bp)	ST	CT	GenBank accession no.	SRA accession no.
S-01	2,928,659	37.9	1,212,448	2,962	78	101	48	244,161	ST1	CT8354	JAGGDY000000000	SRR14027493
S-02	2,890,130	37.9	1,407,694	2,918	79	113	32	1,506,918	ST451	CT8356	JAGGDX000000000	SRR14027492

Whole-genome sequencing (WGS) of the two L. monocytogenes isolates, S-01 and S-02, generated 1,212,448 and 1,407,694 reads, respectively, with mean coverages of 101- to 113-fold and GC contents of 37.9% (Table 1). The NCBI Prokaryotic Genome Annotation Pipeline (PGAP) v5.1 identified, for isolates S-01 and S-02, 2,962 and 2,918 genes, 2,884 and 2,839 coding sequences, 17 and 12 pseudogenes, 78 and 79 RNA genes, 62 and 64 tRNA genes, and 0 and 2 CRISPR arrays, respectively (Table 1). Mash distance analysis (8) and ribosomal multilocus sequence typing (rMLST) (9) identified both isolates as L. monocytogenes. Strains were characterized by multilocus sequence typing (MLST) (10) and core genome MLST (cgMLST) using SeqSphere+ with default settings as described (11). Isolates S-01 and S-02 both had >99.5% good cgMLST targets and were assigned to lineages I and II, serogroups IVb and IIa, sequence type 1 (ST1) and ST451, and cgMLST complex type 8354 (CT8354) and CT8356 (https://www.cgmlst.org), respectively (Table 1).

### Data availability.

The Listeria monocytogenes WGS project has been deposited in DDBJ/ENA/GenBank under BioProject no. PRJNA716154 and accession no. JAGGDY000000000 (S-01) and JAGGDX000000000 (S-02) (first versions). The raw sequence reads have been deposited in the Sequence Read Archive (SRA) under accession no. SRR14027493 (S-01) and SRR14027492 (S-02).
